# Does Adding Dexamethasone to Clomiphene Citrate Improve Ovulation in PCOS Patients? A Triple - Blind Randomized Clinical Trial Study

**Published:** 2011-03-21

**Authors:** Seddigheh Esmaeilzadeh, Masoumeh Golsorkhtabar Amiri, Zahra Basirat, Mahin Shirazi

**Keywords:** PCOS, Clomiphene Citrate, Dexamethasone

## Abstract

**Background:**

A common cause of anovulation is polycystic ovarian syndrome (PCOS). Clomiphene citrate (CC) is the first line of treatment in PCOS patients however approximately 25% of patients may be CC-resistant. This study aimed to evaluate the efficacy of adding dexamethasone (dex) to CC in CC-resistant PCOS patients with the intent to improve ovulation.

**Materials and Methods:**

This randomized controlled trial study was performed on 60 infertile PCOS patients referred to our infertility research center from 2007 to 2009. Patients were randomly divided in two groups and stimulation performed with dex+CC or CC+placebo. Rates of ovulation, pregnancy and number of mature follicles were evaluated.

**Results:**

Ovulation rate in the dex+CC group was 21 out of 30 (70%) and in the CC+placebo group it was 17 out of 30 (56.7%). The pregnancy rate was 5 (16.7%) in the dex+CC group and 3 (10%) in the CC+placebo group. There was no significant difference between rates of ovulation and pregnancy in both groups, but the number of follicles ≥18 mm were significant in the dex+CC group (p<0.05).

**Conclusion:**

Our results showed that addition of dex to CC significantly increased the number of matured follicles, however the ovulation and pregnancy rates were comparable between the two groups (Registeration Number: IRCT 138807041760 N2).

## Introduction

Polycystic ovarian syndrome (PCOS) has multiple reproductive, metabolic and cardiovascular components, with health implications across a woman’s life span (1). Approximately 75% of these women suffer from infertility due to anovulation (2). The first line of treatment to induce ovulation is Clomiphene citrate (CC) (3, 4) but about 20% of CC-treated women that fail to ovulate are considered to be CC-resistant (5) Although ovulation induction with gonadotropin is successful in these patients (6), it is expensive and extensive monitoring is necessary because of the high sensitivity of polycystic ovaries to exogenous gonadotropin, with a high risk of ovarian hyperstimulation, cycle termination, multiple pregnancies and abortion (7). Surgical therapy with laparoscopic ovarian drilling (LOD) may reduce the need for gonadotropins but it is an invasive surgery with complications (8). If anovulation persists or pregnancy does not occur at a dosage level of 150 mg per day, other medications may be added to the regime to induce ovulation (9). There are a few limited adjunctive therapies that can be attempted before gonadotropin therapy or surgical intervention, such as the use of corticosteroids (10). Addition of oral dexamethasone (dex) to clomiphene therapy has been advocated to improve the chances of ovulation and pregnancy (10, 11) without any described side effects or serious sequelae (12). Glucocorticoids may positively affect GnRH pulsatility and increase follicle stimulating hormone (FSH) release. This effect causes the suppression of corticotrophin releasing factor (CRF), which normally suppresses GnRH release. Besides, glucocorticoids reduce the level of circulating adrenal androgens and thus release the ovary from inhibitory androgenic affects (13, 14). Azziz et al. found no differences in ovulatory response to dex therapy between women with and without dehydroepiandrosterone sulfate (DHEAS) excess (15). Parsanezhad et al. have reported improved hormonal levels, follicular development and cumulative pregnancy rates with the addition of dex to CC in CC-resistant patients with PCOS and normal DHEAS (16). Elnashar et al. noted that the mean number of follicles >18 mm at the time of human chorionic gonadotropin (hCG) administration was significantly higher in the dex group (high dose, short course) than the placebo group (17).

There is a lack of studies in Iran on PCOS CCresistant treatment modalities. A number of PCOS CC-resistant patients that have referred to our PCOS clinic in Babol, Northern Iran encouraged us to investigate different types of dex treatment as a cost effective drug.

This study aimed to evaluate the efficacy of adding dex to CC in CC-resistant PCOS patients with the intent to improve ovulation.

## Materials and Methods

A total of 60 infertile women entered this randomized, triple-blind placebo-controlled trial study. Enrolled patients attended the Fatemeh- Zahra Infertility and Reproductive Health Research Center of Babol, Iran from 2008 to 2010. Research Ethics Committee (REC) of Babol University of Medical Science approved this study. Following a description of the study, all patients who agreed to participate signed informed consents. Patients were diagnosed as having PCOS according to the Rotterdam criteria (18). Patients between the ages of 18 and 35 years, with a period of infertility >1/5 years and normal DHEAS levels entered the study. Patients diagnosed with hyperprolactinaemia, or thyroidism, had a pelvic pathology or surgery, or infertility factor other than anovulation were excluded. All patients had previously received CC and were diagnosed as CC-resistant (failure of ovulation after three cycles of CC that reached a dose of 150 mg daily from the third to seventh cycle day). Patients underwent no treatments during the previous three months prior to the dex treatment. Sample size was calculated by S-plus, version 2000 software. Patients were randomly assigned to receive CC and either dex or a placebo using a computergenerated sequence concealed from the study participants. Samples were blinded for the data collector, patients, the doctor and a nurse who administered the drugs.

The two groups were matched for age, duration of infertility and body mass index (BMI). Each patient had only one treatment cycle. All patients underwent induction ovulation as follows: on day 3, each had a baseline ultrasonographic examination (Mylab40, Esaote, Italy). Clomiphene citrate (Iran Hormone, Tehran, Iran), 100 mg, was given from days 3 until 7. Patients were then divided in two equal groups. In addition to CC, from days 5 to 14 of their cycles, each patient was randomly selected to receive the following: i. oral dex (Dexamethasone 0.5 mg, Tamin, Iran), 2 mg/day, in two divided doses (group I), or ii. folic acid 1 mg/day orally as placebo (group II). We choose dex 2.0 mg (high dose) because of the lack of side effects and was more effective than 0.5 mg, according to Beck (11). Transvaginal ultrasound examination was performed the following day after the end of CC and every other day according to follicular size. hCG 10000 IU (Pregnyl; Darou Pakhsh, Iran) was given intramuscularly when at least one follicle measured 16-18 mm. At 24–38 hours after hCG injection, timed intercourse was advised. Two days after receiving hCG, patients were assessed for signs of ovulation (fluid in the cul-de-sac or corpus luteum formation, or disappearance of dominant follicle). Clinical pregnancy was diagnosed when a gestational sac was detected on transvaginal ultrasound examination 25 days after hCG administration. Follicular development, hormonal status, ovulation rate and pregnancy rate were calculated.

### Statistical analysis

Fisher’s exact test, t test, Chi-square and Mann Whitney were used to analyze the data. P value of <0.05 was considered significant.

## Results

This study enrolled 60 PCOS patients. The numbers of participants randomly assigned were 30 in each group. No patients withdrew from the study after randomization.

Rates of ovulation and pregnancy were not significantly higher in the dex + CC group compared to the CC + placebo group, but the mean number of follicles ≥18 mm were significantly higher.

Also, as summarized in table 1, in a comparison of the CC+placebo group and dex+CC group, no significant differences with regards to age, period of infertility, BMI, hirsutism, menstrual regulation and hormonal levels were noted.

Dexamethasone was well tolerated and no patients reported any side effects.

**Table 1:** Demographic criteria and clinical outcomes in CC and dex groups


Variable	CC+Placebo (n=30)	Dex+CC (n=30)

**Age (years)**	23.1 ± 3.45	24.8 ± 3.56
**Duration of infertility (years)**	3.15 ± 1.45	2.98 ± 1.85
**BMI (Kg/m2)**	27.1 ± 2.96	27.56 ± 3.28
**LH (IU/L)**	5.68 ± 3.68	7.07 ± 3.98
**FSH (IU/L)**	4.81 ± 2.64	5.7 ± 3.29
**Irregular Menstruation **	22 (73.3%)	23 (76.7%)
**Hirsutism**	18 (60%)	22 (73.3%)
**Ovulation rate**	17 (56.7%)	21 (70%)
**Number of Follicles >18mm**	0.93 ± 1.04	1.8 ± 1.4*
**Pregnancy rate**	3 (10%)	5 (16.7%)


The values in above table are presented as Mean ± SD and n (%) * Significant (p<0.05)

## Discussion

Addition of dex to CC increased the number of mature follicles in our study but ovulation and pregnancy rates were comparable in the two groups.

In a Parsanezhad et al. study on 230 patients, the ovulation rate was significantly higher in the dex+CC group when compared to the CC alone group. The pregnancy rate was also higher. Both groups were treated for up to six cycles (17). The dose and days of dex in our study was approximately the same as the above study, although their dose of CC was higher (200 mg). Another difference was the sample size and number of treatment cycles. Also, in a study by Elnashar et al. (17), 80 patients were divided in two groups and matched for age, duration of infertility and BMI. The ovulation and pregnancy rates in their dex group showed significant results which were comparable to the Parsanezhad study, although the dose of CC (100 mg) and the treatment days with dex were less (five days) than the current study and Parsanezhad (16). They treated patients for only one cycle, as with our study, but a study by Ashrafi et al. showed no significant statistical difference in the number of retrieved oocytes and transferred embryos in the dex group compared to the placebo group in patients over 35 years of age (19). Hence we are more in agreement with Lord et al. that have suggested that the place for glucocorticoids in therapy as well as CC has yet to be established by further study and is still under discussion. A well-designed randomized controlled trial study is needed to clarify the value of various days of dex therapy in women (20). Further evaluation is needed to clarify whether it is worthwhile to add dex to CC for the stimulation of follicular development, ovulation and pregnancy in CC-resistant PCOS before gonadotropins or not. To clarify, further studies comparing the various regimens (dose, days and treatment cycles of dex) are required. One limitation to our study was the lack of progesterone measurements to assist with confirmation of ovulation.

## Conclusion

Addition of dex to CC enhances the number of mature follicles significantly but the ovulation and pregnancy rate is comparable to CC alone.
